# Pulmonary Infiltrates and Eosinophilia in a 25-Year-Old Traveler

**DOI:** 10.1371/journal.pntd.0002201

**Published:** 2013-06-13

**Authors:** Jose Muñoz, Edelweiss Aldasoro, Maria Jesús Pinazo, Pedro Arguis, Joaquim Gascon

**Affiliations:** 1 Barcelona Centre for International Health Research (CRESIB, Hospital Clínic-Universitat de Barcelona), Barcelona, Spain; 2 Radiology Department, Hospital Clinic, Barcelona, Spain; (The George Washington University Medical Center, United States of America)

## Question

**Figure 1:**
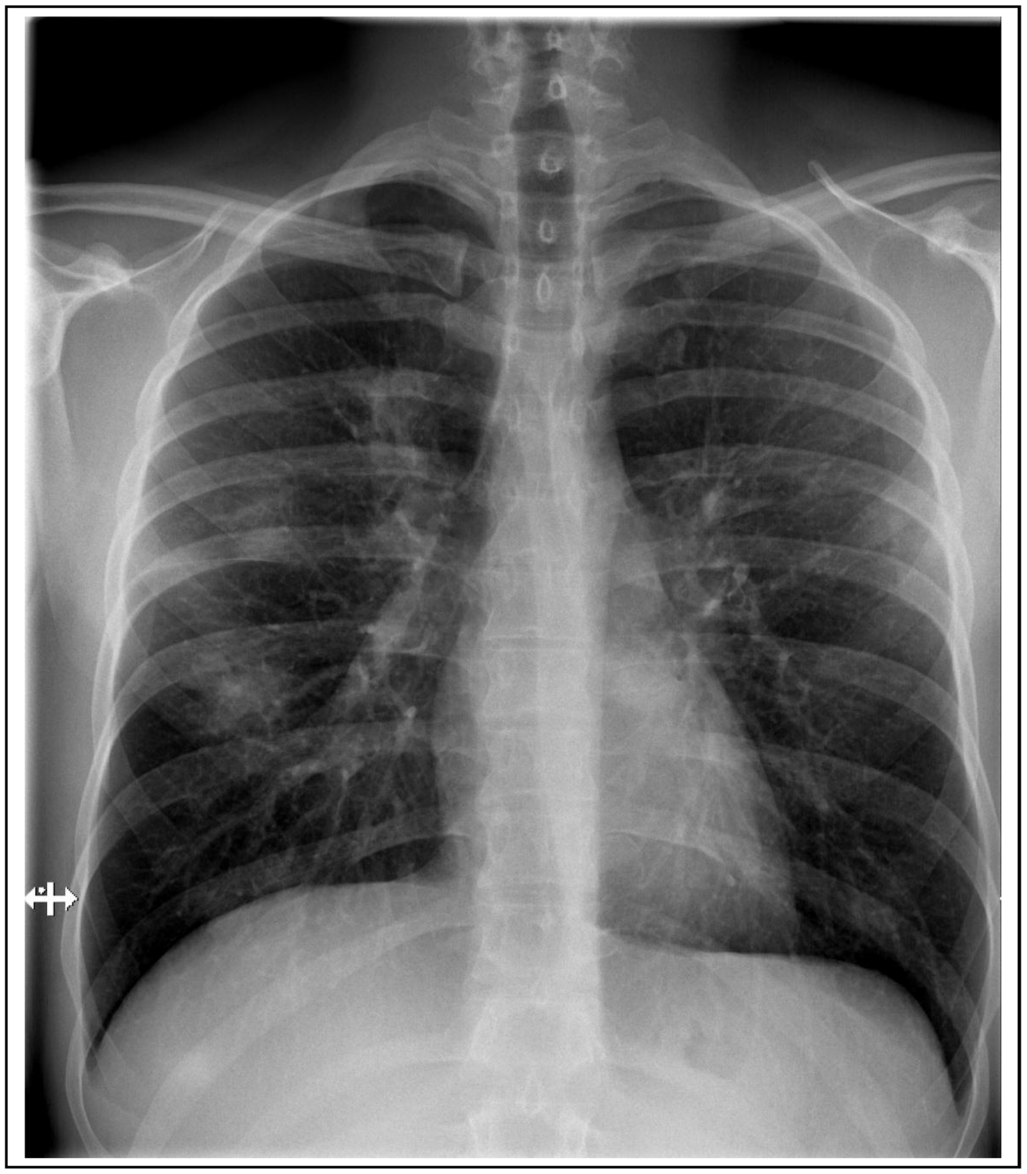
Chest X-ray showing patchy infiltrates in both lungs.

**Figure 2:**
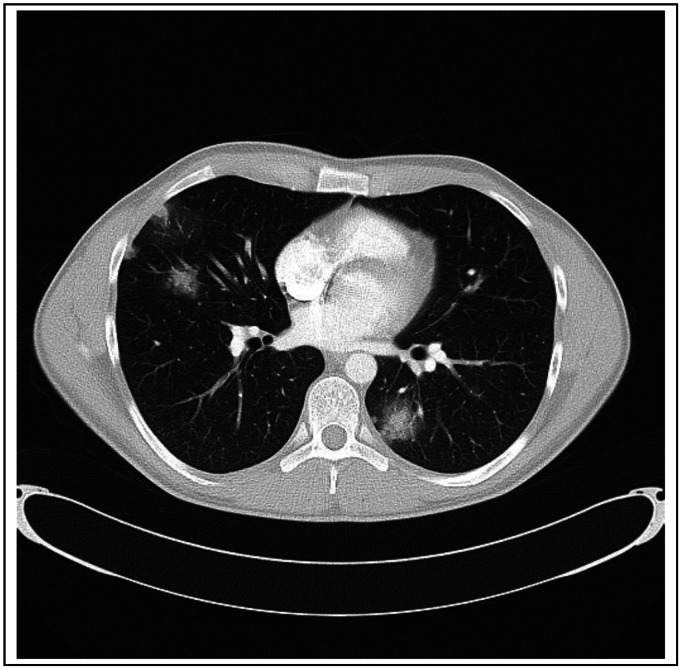
Multiple peripheral bilateral pseudonodular lesions with surrounding ground-glass-opacity halo in CT thorax scan.

A 25-year-old Spanish male travelled to Senegal in September 2009, where he swam near the Dindefelo fresh-water falls. Five weeks later, he presented with fever, myalgia, and dry cough. His complete blood count showed a hemoglobin level of 157 g/L, platelet count of 123.000 platelets/µL, and a leukocyte count of 8.670 cells/µL, with 9% eosinophils. Malaria smear, blood cultures, and serologies for common viral and bacterial infections were negative. Titers of an indirect hemagglutination test for *Schistosoma mansoni* were 1∶80. The patient was treated with a single dose of praziquantel (40 mg/kg) and prednisone (30 mg) for three days. After treatment, the dry cough increased and he developed moderate dyspnea, with increasing malaise and myalgia. A second blood sample revealed eosinophilia of 1.200 cells/µL. A chest X-ray showed patchy infiltrates in both lungs (Figure 1), and a CT scan showed multiple peripheral bilateral pseudonodular lesions with surrounding ground-glass-opacity halo (Figure 2).

The patient gave consent to have his case details published.

## Diagnosis

### Acute pulmonary schistosomiasis

The patient was treated with prednisone (1 mg/kg/day) for seven days and became asymptomatic within 48 hours. A repeat chest X-ray 24 hours later showed no abnormalities.

He was treated again with praziquantel (40 mg/kg/day) for three days, three months after initial diagnosis. Antibodies against *Schistosoma* species increased in a control blood sample six months later (titers 1∶160). No *Schistosoma* eggs were found in the four samples of stools and urine tested.

The patient remained asymptomatic up to 24 months after treatment.

## Discussion

Infection with *Schistosoma* species is acquired by exposure to fresh water that contains cercariae, the infective form of this trematode [1]. The infection is highly endemic in sub-Saharan Africa, and causes chronic disease with urogenital, intestinal, or hepatic manifestation in most cases [2], [3].

Acute schistosomiasis or Katayama syndrome is an early manifestation of the infection and may occur between two and 12 weeks after primary infection. It is considered to be a toxemic syndrome due to a hypersensitivity reaction to the migrating and maturing larvae of the parasite and its eggs [4], [5]. It can be seen in nonimmune individuals exposed to an infected source of fresh water. Therefore, acute schistosomiasis is rarely diagnosed in endemic areas, but frequently observed after infection of naive travelers to endemic countries, and after infection of naive populations where the disease is emerging (e.g., due to industrialization and construction of dams) [4].

Symptoms of acute schistosomiasis usually involve fever, malaise, and high-grade eosinophilia (usually over 3,000 eosinophils 10^9^/L) [4], [5]. Although eosinophilia has been considered a constant finding, it is only observed in about 70–80% of cases [6]. Thus, the absence of eosinophilia does not exclude Katayama syndrome. Other possible symptoms are diarrhoea, urticaria, edema, hepatosplenomegaly, and cough. Usually the disease is self-limited, although some patients suffer severe manifestations and may need hospitalization [1], [4], [5].

Although not frequently described, pulmonary involvement with *Schistosoma* can occur in acute cases and may be much more common than previously suspected [7], [8]. Dry cough is a frequent symptom in patients with Katayama syndrome, but shortness of breath, dyspnea, wheeze, and chest pain may also appear [3], [7], [9]. Chest radiographs may reveal nodular lesions with ill-defined borders and less frequently pleural effusion or reticulonodular infiltrates [7], [9]. The most common findings when performing a chest CT scan are pulmonary nodules from 2 to 15 mm, and ground-glass attenuation areas, as found in our case. The histopathological translation of these radiological images may be an interstitial eosinophilic pneumonitis and immune complex deposition of the lung parenchyma [10], [11].

Diagnosis of acute schistosomiasis in travelers remains challenging, and is often done in retrospect after seroconversion, since in most cases serological tests and stool examination first become positive several weeks after exposure [5].

Praziquantel is the drug of choice to treat all the species of *Schistosoma* [2]. However, its use is not necessarily effective in the acute phase of the infection since it has less impact on the immature worms [5]. In addition, clinical worsening of patients has been described after treatment, possibly due to an allergic response to the parasite killing, especially when no steroids are given concomitantly [5]. These side effects may occur and are not a reason to refuse treatment. The respiratory symptoms of our patient worsened after treatment with praziquantel and steroids: this could be a treatment effect but may also be provoked by a paradoxical reaction that is described in patients with Katayama syndrome, even in the absence of any treatment [5]. Since acute schistosomiasis is considered to be a hypersensitivity reaction, treatment with corticosteroids should be taken into account, especially in cases with severe manifestations [5], [12].

The use of praziquantel in this acute phase does not preclude from repeating the treatment two to three months after the acute phase when all parasites have matured and are susceptible to praziquantel [2], [4].

**Key Learning Points**

- Timely diagnosis of acute schistosomiasis still relies on clinical and epidemiological exposure history.
- Even though dry cough is commonly found in cases of acute schistosomiasis, severe respiratory complications might also occur.
- Nodular lesions with ill-defined borders in chest X-ray and pulmonary nodules with ground-glass attenuation areas in CT scan are the most common radiological manifestations of acute pulmonary schistosomiasis.
- Optimal treatment of acute schistosomiasis is still unclear.
- Symptoms might worsen after treatment with praziquantel. The concomitant use of steroids is highly recommended.

## References

[1] Corachan M (2002) Schistosomiasis and international travel. Clin Infect Dis 35: 446–450. 12145730 10.1086/341895

[2] Gryseels B, Polman K, Clerinx J, Kestens L (2006) Human schistosomiasis. Lancet 368: 1106–1118. 16997665 10.1016/S0140-6736(06)69440-3

[3] Meltzer E, Artom G, Marva E, Assous MV, Rahav G, et al (2006) Schistosomiasis among travelers: new aspects of an old disease. Emerg Infect Dis 12: 1696–1700. 17283619 10.3201/eid1211.060340 PMC3372337

[4] Ross AG, Vickers D, Olds GR, Shah SM, McManus DP (2007) Katayama syndrome. Lancet Infect Dis 7: 218–224. 17317603 10.1016/S1473-3099(07)70053-1

[5] Jauréguiberry S, Paris L, Caumes E (2010) Acute schistosomiasis, a diagnostic and therapeutic challenge. Clin Microbiol Infect 16: 225–231. 20222897 10.1111/j.1469-0691.2009.03131.x

[6] Agbessi CA, Bourvis N, Fromentin M, Jaspard M, Teboul F, et al (2006) Acute schistosomiasis in French travellers. Rev Med Interne 27: 595–599. 16822596 10.1016/j.revmed.2006.05.006

[7] Schwartz E, Rozenman J, Perelman M (2000) Pulmonary manifestations of early schistosome infection among nonimmune travelers. Am J Med 109: 718–722. 11137487 10.1016/s0002-9343(00)00619-7

[8] Schwartz E (2002) Pulmonary schistosomiasis. Clin Chest Med 23: 433–443. 12092037 10.1016/s0272-5231(01)00013-2

[9] Lambertucci JR, Silva LC, de Queiroz LC (2007) Pulmonary nodules and pleural effusion in the acute phase of schistosomiasis mansoni. Rev Soc Bras Med Trop 40: 374–375. 17653482 10.1590/s0037-86822007000300027

[10] Nguyen LQ, Estrella J, Jett EA, Grunvald EL, Nicholson L, et al (2006) Acute schistosomiasis in nonimmune travelers: chest CT findings in 10 patients. AJR Am J Roentgenol 186: 1300–1303. 16632722 10.2214/AJR.05.0213

[11] Souza Sdos S, Souza Neto JP, Andrade ZA (2009) Pulmonary changes during acute experimental murine mansoni schistosomiasis. Rev Soc Bras Med Trop 42: 5–8. 19287927 10.1590/s0037-86822009000100002

[12] Leshem E, Maor Y, Meltzer E, Assous M, Schwartz E (2008) Acute schistosomiasis outbreak: clinical features and economic impact. Clin Infect Dis 47: 1499–1506. 18990059 10.1086/593191

